# Pesticide effects on the abundance of springtails and mites in field mesocosms at an agricultural site

**DOI:** 10.1007/s10646-022-02599-3

**Published:** 2022-11-01

**Authors:** Heidi Sjursen Konestabo, Tone Birkemoe, Hans Petter Leinaas, Cornelis A. M. van Gestel, Sagnik Sengupta, Katrine Borgå

**Affiliations:** 1 Department of Biosciences, University of Oslo, Oslo, Norway; 2 The Science Library, University of Oslo, Oslo, Norway; 3 Faculty of Environmental Sciences and Natural Resource Management, Norwegian University of Life Sciences, Oslo, Norway; 4 Amsterdam Institute for Life and Environment (A-LIFE), Faculty of Science, Vrije Universiteit, Amsterdam, The Netherlands

**Keywords:** Imidacloprid, Neonicotinoids, Soil fauna, Acari, Collembola, Decomposers

## Abstract

The use of pesticides to protect crops often affects non-target organisms vital to ecosystem functioning. A functional soil mesofauna is important for decomposition and nutrient cycling processes in agricultural soils, which generally have low biodiversity. To assess pesticide effects on natural soil communities we enclosed intact soil cores in situ in an agricultural field in 5 cm wide mesocosms. We used two types of mesh lids on the mesocosms, allowing or preventing migration of mesofauna. The mesocosms were exposed to the insecticide imidacloprid (0, 0.1, 1, and 10 mg/kg dry soil) and left in the field for 20 days. Overall, regardless of lid type, mesocosm enclosure did not affect springtail or mite abundances during the experiment when compared with undisturbed soil. Imidacloprid exposure reduced the abundance of both surface- and soil-living springtails in a concentration-dependent manner, by 65–90% at the two highest concentrations, and 21–23% at 0.1 mg/kg, a concentration found in some agricultural soils after pesticide application. Surface-living springtails were more affected by imidacloprid exposure than soil-living ones. In contrast, neither predatory nor saprotrophic mites showed imidacloprid-dependent changes in abundance, concurring with previous findings indicating that mites are generally less sensitive to neonicotinoids than other soil organisms. The possibility to migrate did not affect the springtail or mite abundance responses to imidacloprid. We show that under realistic exposure concentrations in the field, soil arthropod community composition and abundance can be substantially altered in an organism-dependent manner, thus affecting the soil community diversity.

## Introduction

Agriculture has been identified as a major contributor to biodiversity loss, due to increasing land area use as well as the physical and chemical manipulations involved in conventional farming. Agricultural practices such as tillage, monoculture, drainage, and the use of pesticides to protect crops from herbivory and disease often have adverse effects on organisms vital to ecosystem functioning, including soil biota (McLaughlin and Mineau, 1995; Stoate et al., 2009; Thiele-Bruhn et al., 2012; Tsiafouli et al., 2015). Soil biota contribute to ecosystem functioning by their involvement in processes such as decomposition and nutrient cycling. Agricultural soils are generally low in biodiversity (Tsiafouli et al., 2015), which makes it even more important to maintain the soil organisms and their functions.

In ecotoxicological hazard assessment of chemicals, effects of toxicants on single organisms are assessed under optimal laboratory conditions to isolate the effects of the toxicant, using standardized model species that are particularly well suited to keep in laboratory cultures, often with an acute exposure time of 28 days (e.g., OECD, 2016). While such standardized tests are useful for assessing toxicity of specific substances and their specific modes of action, the tests are not sufficient for predicting the impact on populations in agricultural fields or natural ecosystems, where interaction with environmental conditions and other organisms occur in a complex manner. Such interactions may lead to unexpected results (Lindenmayer et al., 2010; Paine et al., 1998) and are therefore of great ecological relevance. In outdoor field systems, organisms may also survive pesticide application in refuges with lower concentrations, or they may recolonize from the surroundings as the pesticide degrades. Even with a uniform application of a pesticide, the distribution in the litter and soil can be uneven due to leaching, organic carbon content, and the dynamic nature of the soil matrix (Cox et al., 1998; Mörtl et al., 2016; Ondrasek, 2019; Selim et al., 2010; Zhang et al., 2018). These important mechanisms of ecosystem variability cannot be easily evaluated in the laboratory. Field studies offer the opportunity of measuring the effects of environmental toxicants in natural communities with ecologically important and naturally abundant species, and thus evaluate biological responses under realistic scenarios (Rudén et al., 2017). It is still largely unknown how differential effects and altered interactions among organisms in the field after toxicant exposure may change ecosystem services (Awuah et al., 2020). Simplified field experiments using intact soil core mesocosms may offer the opportunity to study responses of the soil community while controlling some of the variations.

The agricultural pesticide imidacloprid was one of the most frequently used neonicotinoid insecticides in Europe in the past decades. Although imidacloprid from 2018 is restricted from use in agriculture in the EU, it is still used in indoor greenhouses (EFSA, 2016) and in veterinary medicine (Wells and Collins, 2022). Imidacloprid is also still widely used in other parts of the world (e.g., Nunes et al., 2021; US EPA, 2020). It is highly effective against a wide array of herbivorous insects, has low toxicity to vertebrates, and its high persistence and mobility (Bonmatin et al., 2015; Zhang et al., 2018) make it suitable for seed dressing applications (Jeschke et al., 2011). As a result, a significant part of the applied dose stays or ends up in the soil. The half-life varies between studies using different soil types as well as between studies with similar soil types, with a reported range of 28–1250 days (Goulson, 2013). The high persistency is confirmed by the reports on imidacloprid residues present in topsoil samples originating from all European regions and across 6 different crop classes, in a review of European agricultural soils (Silva et al., 2019).

There are numerous records of adverse effects of imidacloprid exposure in laboratory conditions on pollinating insects and soil fauna as well as in aquatic ecosystems (e.g., Alves et al., 2013; Blacquiere et al., 2012; de Lima e Silva et al., 2017, 2021; Goulson, 2013; Peck, 2009; van Gestel et al. 2017). For the springtail *Folsomia candida*, a common laboratory model species, the median lethal concentration (LC50) is ~0.5 mg imidacloprid/kg dry soil, while the soil mite *Oppia nitens* and the soil oligochaete *Enchytraeus crypticus* show a lower sensitivity with LC50s of 360 and >30 mg/kg dry soil, respectively (de Lima e Silva et al., 2017, 2020). In the surface-living springtail *Hypogastrura viatica*, an LC50 was recently estimated to 0.17 mg/kg (Kristiansen et al. 2021), a concentration comparable to the soil concentrations reported by Silva et al. 2019. This could imply large possible effects on soil fauna not yet investigated, as well as soil fauna in natural conditions.

The main aim of the present study is to assess how pesticide exposure affects a soil community using a method applying intact soil mesocosms in-situ, to understand community-level responses to a pesticide under field conditions. We address this by asking whether (1) imidacloprid exposure affects the soil mesofauna abundance in a concentration- and organism type-related manner, and (2) whether this effect is modulated by the possibility of the mesofauna to migrate into and out of the mesocosms. We conducted a 20-day field experiment with in-situ mesocosms containing intact soil cores exposed to imidacloprid. We focused on major soil mesofauna groups of springtails and mites. We expected that the abundance of springtails would decrease with increasing concentrations of imidacloprid, whereas that of mites would remain unaffected at low concentrations. We also expected differences between major groups of springtails and mites related to their microhabitat preferences and dispersal abilities.

## Methods

### Field site and treatments

Our mesocosm experiment was designed to mimic the pesticide concentrations found in soil based on past use, by performing a short-term study at the end of the season. The pesticide exposure in mesocosms was conducted from September 20^th^ to October 9^th^, 2018, in a grass pasture at Kjerringjordet in Ås, Norway (59°39′N, 10°45′E). The site was previously used as an agricultural field with grass vegetation and no pesticide use. The site was mowed one week before starting the experiment. Treatments consisted of four concentrations (0, 0.1, 1, 10 mg/kg) of imidacloprid in mesocosms with intact soil cores including litter layer and soil fauna, with two different types of lids. We chose a range of concentrations of imidacloprid based on estimated predicted environmental concentrations (PECs) ranging from of 0.04–0.24 mg/kg from a single application of imidacloprid, calculated based on recommended doses provided by the pesticide producers (Bandeira et al., 2020; de Lima e Silva et al., 2020). We also considered LC50s in springtails found in previous studies under laboratory conditions (de Lima e Silva et al., 2017, 2020, 2021; Kristiansen et al., 2021), and included concentrations well above reported LC50s to take into account the supposed low susceptibility of mites to imidacloprid (de Lima e Silva et al., 2017). Two types of lids were chosen to evaluate if soil fauna migration into or out of the exposed area affected the overall differences in abundances of springtails and mites depending on treatment. Therefore, lids with large or small mesh sizes were used, referred to as “open” and “closed”, respectively. It was, however, not an experiment of animal behaviour as such, which would require observational studies. During the experiment, there was a daily average precipitation of 2.02 mm, with a total precipitation of 40.4 mm, and an average daily air temperature of 8.4 °C (data from https://www.met.no/).

### Experimental setup

**Fig. 1:**
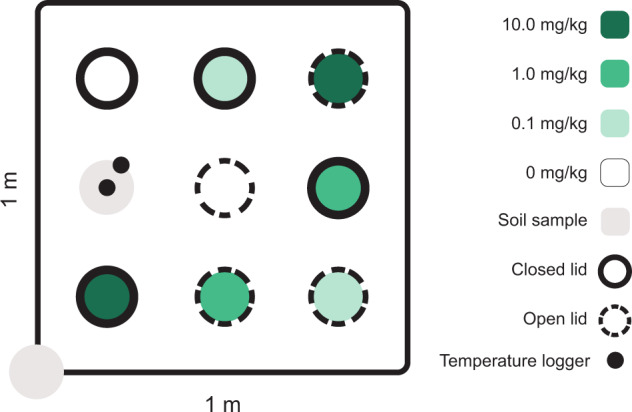
Schematic set up of one block (replicate, block *n* = 6) of the field experiment with mesocosms exposed to imidacloprid. Each block was 1 × 1 m, and each mesocosm/soil sample diameter was 5 cm. Note that the circles indicating mesocosms/soil samples are not drawn to scale for clarity. Mesocosms were exposed to four concentrations of imidacloprid (0, 0.1, 1 and 10 mg/kg dry soil) indicated by different shadings of green, and there were two types of mesocosm lids to allow or limit migration. Open lids that facilitated migration had a mesh size of 1.5 mm (dashed lines), closed lids that prevented migration had a mesh size of 0.3 mm (solid lines). One pretreatment, undisturbed soil sample was taken at the start (grey circle within the block) and another un-treated, undisturbed soil sample at the end of the experiment (grey circle at the edge of the block) for estimation of soil fauna abundance in soil without mesocosms. The empty position was replaced by a mesocosm filled with a soil core from the area adjacent to the block and used for temperature measurements. Two temperature loggers (small black dots) were placed—one inside and one outside this mesocosm

Six 1 ×1 m blocks were chosen with 1 m distance between blocks across the field. Within each replicate block, we installed four open and four closed mesocosms randomly placed in a 3 × 3 grid (Fig. 1), with at least 10 cm distance between mesocosms. The mesocosms consisted of a plastic cylinder (5 cm Ø, 5 cm height) with a metal mesh bottom (0.3 mm mesh size) to allow water drainage. Half the mesocosms had open lids with a mesh size of 1.5 mm, allowing mesofauna to move in and out while holding the soil core intact with litter (Leinaas et al., 2015) and preventing disturbance by larger animals. The other half of the mesocosms had closed lids with a mesh size of 0.3 mm, preventing migration of mesofauna while allowing water and gas exchange.

Imidacloprid was applied at the soil surface of the mesocosm before placing the lid, simulating common agricultural pesticide application practices. Imidacloprid treatments of 0, 0.1, 1, or 10 mg/kg were randomly assigned to both the closed and open mesocosms within each replicate block. An estimate of soil fauna abundance at the start of the experiment was done by taking an undisturbed sample from each block (Fig. 1). After removing this soil sample, a mesocosm containing a soil core from the area adjacent to the block was installed at the position where the undisturbed soil sample had been removed. One temperature logger (iButtons, Maxim Integrated Products, Inc.) was placed inside and one outside of this mesocosm, 1 cm into the soil, to register temperature throughout the experiment. Three mesocosms with temperature loggers had open lids, and three had closed lids.

### Imidacloprid solution

To minimize the handling time of the mesocosms, we estimated the average dry weight of soil in a mesocosm based on 8 soil samples from the field site. The imidacloprid concentrations in the dosing solutions were calculated based on the average sample dry soil weight, and assuming a homogenous distribution of the applied dose over the entire soil mass in the mesocosm. Imidacloprid concentrations were prepared by dissolving imidacloprid powder (CAS No. 138261-41-3, Sigma-Aldrich) in distilled water to the highest concentration and diluting to the target concentrations. For the control (0 mg/kg imidacloprid), we used distilled water.

### Installation of mesocosms and imidacloprid application

The mesocosms were installed by taking a 5 cm deep soil sample using a metal soil corer with the same diameter as the mesocosm (5 cm) and placing the intact soil core into the mesocosm. The plant layer was cut down to approximately 0.5 cm height, and the litter layer was kept in the sample. Imidacloprid was added using a pipette with 2 mL imidacloprid solution corresponding to the different treatments, corresponding to 1 mm of water layer for the surface area of the mesocosms. The imidacloprid solution was drip applied evenly to the soil surface, under the plant and litter layer, and the lid (open or closed) was placed. The mesocosm was then installed into the soil where the sample was taken.

### Sampling and identification

The undisturbed soil sample collected at the start of the experiment was transported to the laboratory at the University of Oslo for immediate analysis as described below. After 20 days, all mesocosms and an additional undisturbed soil sample from each block were collected and transported to the laboratory for analysis. The temperature loggers were retrieved, and data downloaded (OneWireViewer, Maxim Integrated Products, Inc).

Mesocosms and undisturbed soil samples were weighed and transferred to an extraction system with a temperature gradient (modified Tullgren funnel) to extract active springtails and mites, as described by Konestabo et al. (2020). Extracted specimens were collected in benzoic acid and transferred to glycerol for counting and identification. Extraction lasted for 1 week with a temperature increase from 20 °C to 60 °C, to gradually evaporate all the water in the soil samples. By re-weighing the soil samples the moisture content and dry soil weight was calculated.

Springtails were identified to family, genus or species (Fjellberg 1998, 2007) and subsequently divided into two groups based on life form classification following Hopkin (1997), reflecting their vertical distribution in the soil. Our group of surface-living springtails included epedaphic and hemiedaphic species (living on the surface or in the litter, respectively). In our sampled material this comprised the springtail families Hypogastruridae, Isotomidae, Entomobryidae and Symphypleona. The soil-living springtails consisted of eu-edaphic species including only Onychiuridae in our samples (see Supplementary information, Table S1). The reason for this grouping was that we expected that the greatest difference in treatment exposure would be between the species living in the soil and those with a distribution near the surface. Mites were identified to order or family (Krantz and Walter, 2009) and subsequently divided into functional groups based on their feeding ecology, as saprotrophs or predators.

### Statistical analyses

All statistical analyses were performed using R (R Core Team, 2020). To investigate the effects of imidacloprid treatments and migration possibility (open or closed lid) on springtail and mite abundance, we counted the number of springtails or mites from the treatments and compared them to imidacloprid controls (concentration = 0 mg/kg). We fitted log-linear mixed effects models to abundance data (number of animals +1), with imidacloprid concentration and lid type as fixed effects including their interaction, and with block as random effect, using the package *nlme* (Pinheiro et al., 2020). Relative differences (i.e., percentage change relative to the control) were computed directly from the exponents of linear contrasts. Total variance explained by the models (conditional R^2^) were calculated according to the method described by Nakagawa and Schielzeth (2013), as implemented in the package *MuMIn* (Bartoń, 2022). Statistical power of the models were computed using the function *pwr.f2.test* in the package *pwr* (Champely et al., 2020). Model selection and validation was done according to Zuur et al. (2009), and model assumptions were validated by graphical investigation of residual plots.

Several dose-response functions were compared using the *mselect()* function in the *drc* package (Ritz et al., 2015). The selected model was fitted to the abundance data with nominal imidacloprid concentration as fixed effect. Median effect concentration (EC50) values were calculated from the selected models.

To ensure that the mesocosms themselves did not affect the springtail and mite abundance, a log-linear model was fitted to abundance data (number of animals +1) from the control samples (imidacloprid concentration = 0 mg/kg) with open and closed lids, and compared to undisturbed soil samples taken at the end of the experiment. Relative differences (percentage change relative to control) were calculated as described above.

## Results

### Effects of imidacloprid exposure and migration possibility on abundance

#### Springtails

At the two highest concentrations of imidacloprid, the total springtail abundance was lower than in the controls, with 73 and 90% reduction at 1.0 and 10 mg/kg, respectively (Table 1). The possibility to migrate (lid effect) did not modify these abundance effects significantly as identified by overlapping confidence intervals. Thus, there was a concentration-related reduction in the springtail abundance, although with large variation between samples. Estimated EC50 for the effect on total springtail abundance was 0.30 (CI: −0.17, 0.77) mg imidacloprid/kg dry soil.

**Table 1:** Summary of the effects of imidacloprid treatments (percent change compared to the control) and migration possibility (lid type) on the abundance of springtails and mites in field mesocosms estimated from log-linear mixed effect models

	Effect size (%)				Variance explained	
Functional group	0.1 mg/kg	1.0 mg/kg	10.0 mg/kg	Open lid	cR^2^	Power
All springtails	−22.4 (−60.5, 52.2)	−**72.7** (−**86.1**, −**46.5)**	−**89.8** (−**94.8**, −**79.9)**	26.6 (−21.4, 103)	0.56	0.99
Surface-living springtails	−23.3 (−60.3, 48.2)	−**71.2** (−**85.1**, −**44.4)**	−**90.2** (−**94.9**, −**81.1)**	28.1 (−19.6, 104)	0.58	0.99
Soil-living springtails	−21.7 (−62.2, 61.2)	−**81.0** (−**90.8**, −**60.7)**	−**65.9** (−**83.5**, −**29.6)**	31.3 (−21.5, 119)	0.38	0.99
All mites	30.2 (−17.7, 105)	10.5 (−29.3, 72.6)	47.1 (−6.2, 130)	3.0 (−22.1, 36.0)	0.09	0.34
Saprotrophic mites	28.2 (−19.4, 103)	8.9 (−30.8, 71.4)	46.6 (−8.4, 134)	−0.3 (−25.8, 34.0)	0.08	0.30
Predatory mites	29.3 (−17.0, 101)	6.5 (−31.6, 65.8)	25.8 (−19.2, 95,8)	19.3 (−12.8, 63.1)	0.07	0.26

Effect sizes (median and 95% confidence interval) of the imidacloprid treatments (concentration in mg/kg dry soil) and open lid with migration possibility is expressed as percentage change in abundance relative to the controls or the closed lid without migration possibility. When the 95% confidence interval is not including zero, it is significantly different from controls, and is highlighted in bold. The values in each row have been computed from separate log-linear mixed effects models fitted to the different functional groups of soil fauna with imidacloprid concentration and lid type as fixed effects and block as random factor. The total variance explained (cR^2^ = conditional R^2^) and statistical power of each model are shown

**Fig. 2:**
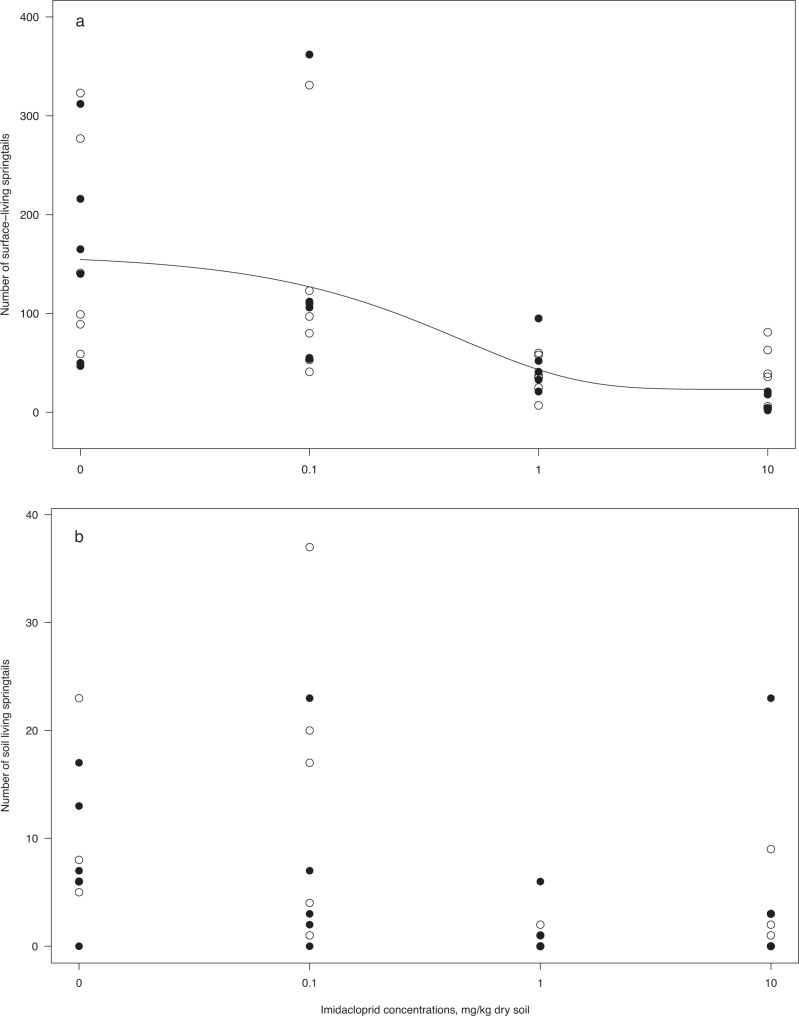
Abundance (number of individuals) of (**a**) surface-living springtails and (**b**) soil-living springtails exposed to imidacloprid in field mesocosms under different conditions of migration possibilities. Dots show the numbers of springtails in mesocosms with open lids (open circles) and closed lids (filled circles), *n* = 6. The line shows the fit of a three-parameter dose-response model and the shaded area the 95% confidence interval, *n* = 12 (pooled open and closed mesocosm lids)

Higher imidacloprid concentrations reduced the abundance of both surface-living and soil-living springtails, with the concentration-dependent reduction in abundance being more evident in the surface-living springtails, which showed little overlap in the 95% CIs at 1 and 10 mg/kg imidacloprid (Table 1). The response of soil-living springtails to imidacloprid did not follow a clear concentration-related decline owing to overlapping and rather wide 95% CI at 1 and 10 mg/kg. At the 1 mg/kg treatment, the 95% CIs of the surface-living and soil-living springtails showed substantial overlap, whereas a small overlap was seen at 10 mg/kg, suggesting that the surface-living springtails are more sensitive to imidacloprid exposure (Table 1). Neither springtail group was affected by the possibility to migrate (lid effect), thus the interaction effects (lid*concentration) were dropped from the model based on AIC model validation according to Zuur et al. (2009). A concentration-response model satisfactorily fitted the abundance of surface-living springtails (Fig. 2a), but did not capture the high variation in the abundance of soil-living springtails (Fig. 2b). The EC50 for effects of imidacloprid on surface-living springtails was similar to that of all springtails (EC50 = 0.30, CI: −0.18, 0.78 mg/kg). The total variance in abundance of springtails explained by the models in the power analyses (conditional R^2^) is shown in Table 1.

#### Mites

**Fig. 3:**
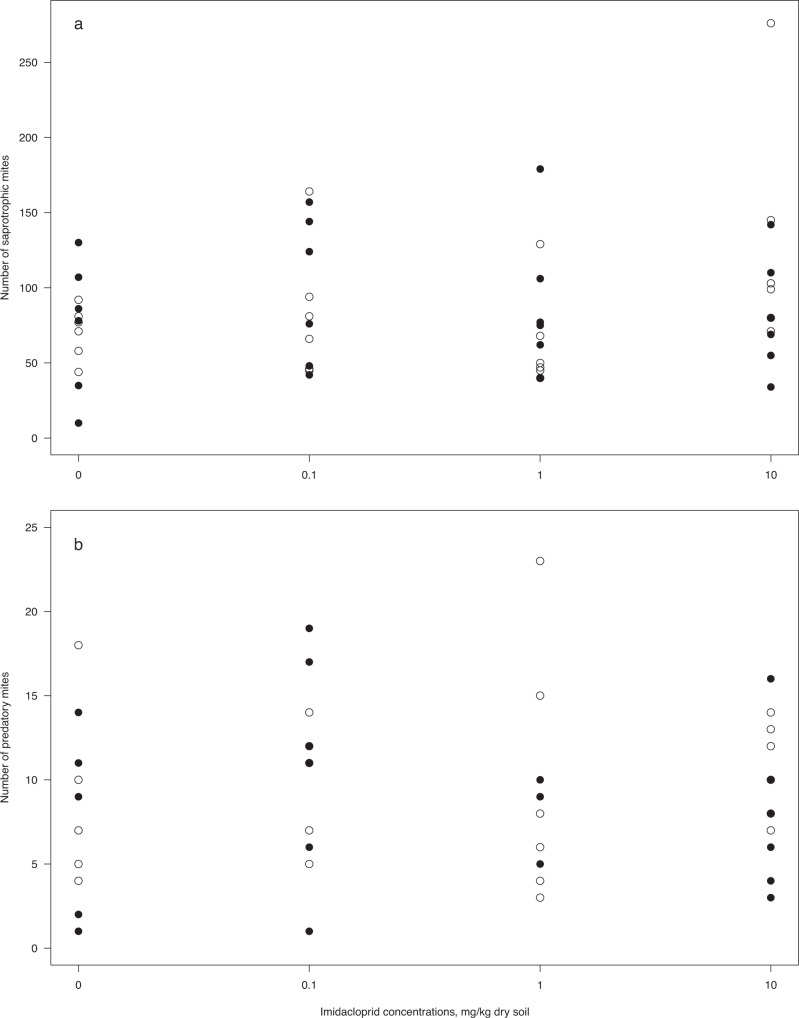
Abundance (number of individuals) of (**a**) saprotrophic mites and (**b**) predatory mites exposed to imidacloprid in field mesocosms under different conditions of migration possibilities. Dots show the numbers of mites in mesocosms with open lids (open circles) and closed lids (filled circles), *n* = 6

The abundance of mites, both total number of mites and the number of saprotrophic and predatory mites, was not affected by imidacloprid exposure nor migration possibility, with overlapping confidence intervals for all comparisons (Table 1, Fig. 3). There was an overall higher abundance of saprotrophic mites (Oribatida and Prostigmata, Fig. 3a) than predatory mites (Fig. 3b). The total variance explained by the models in the power analyses (conditional R^2^) is shown in Table 1.

### Effects of mesocosm enclosure

**Fig. 4:**
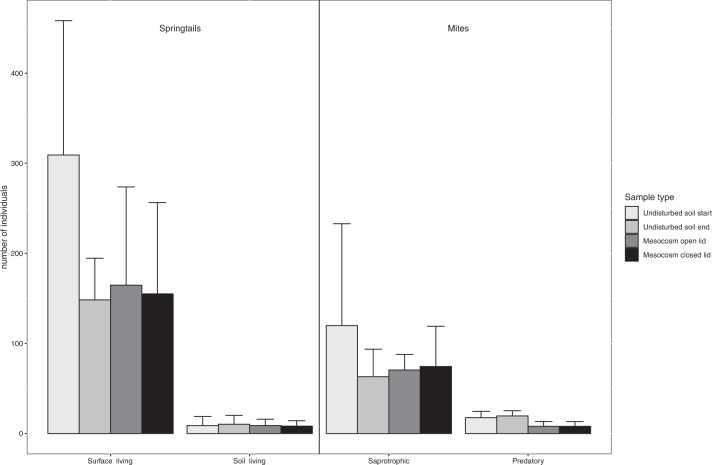
Average number of springtails and mites (+ standard error) from control mesocosms without imidacloprid added, and undisturbed soil samples with no mesocosms. The mesocosms were incubated in an agricultural field for 20 days. The undisturbed soil samples were taken at the start and end of the experiment. Four different sample types were compared: Undisturbed soil start = Undisturbed soil samples taken at the start of the experiment, Undisturbed soil end = Undisturbed soil samples taken at the end of the experiment, Mesocosm open lid = Mesocosms with lids with mesh size 1.5 mm, no imidacloprid, Mesocosm closed lid = Mesocosms with lids with mesh size 0.3 mm, no imidacloprid. *n* = 6

Incubating the soil in mesocosms did not affect the abundance of neither surface- nor soil-living springtails when comparing control samples (imidacloprid concentration = 0 mg/kg, open and closed lids) with undisturbed soil at the end of the experiment (Fig. 4, Table 2). Likewise, saprotrophic mite abundance was unaffected by mesocosm enclosure. However, the abundance of predatory mites was higher than in the undisturbed soil samples (*p* = 0.02, Fig. 4, Table 2). All effects were independent of lid type. An overview of the total number of springtails and mites sampled at the site can be found in Table S1 in the Supplementary information.

**Table 2:** Summary of the effects of mesocosms with and without migration possibilities (percent change compared to the undisturbed samples) on the abundance of springtails and mites estimated from log-linear regression models

	Effect size (%)		Variance explained	
Functional group	Mesocosms with open lids	Mesocosms with closed lids	cR^2^	Power
All springtails	−4.5 (−53.6, 96.5)	−11.5 (−56.9, 82.2)	0.007	0.06
Surface-living springtails	−3.7 (−54.7, 105)	−12.5 (−58.9, 86.5)	0.009	0.06
Soil-living springtails	−1.7 (−62.7, 177)	−18.8 (−70.2, 121)	0.02	0.07
All mites	−0.4 (−52.7, 110)	−18.0 (−61.1, 72.6)	0.02	0.07
Saprotrophic mites	19.1 (−44.2, 155)	−0.5 (−53.4, 113)	0.02	0.07
Predatory mites	−**59.8** (−**80.8**, −**15.9)**	−**64.0** (−**82.8**, −**24.7)**	0.38	0.78

Effect sizes (median and 95% confidence interval) of the different mesocosm types (with open lids allowing migration, and with closed lids preventing migration) is expressed as percentage change in abundance relative to the undisturbed controls. When the 95% confidence interval is not including zero, it is significantly different from controls, and is highlighted in bold. The values in each row have been computed from separate log-linear regression models fitted to the different functional groups of soil fauna with sample type as fixed effect. The total variance explained (cR^2^ = conditional R^2^) and statistical power of each model are shown

There was a slightly higher variation in temperatures measured outside the mesocosms than inside, with higher temperatures during the day and lower temperatures during the night (Supplementary information, Fig. S1). However, the daily averages showed very little difference, with only a slightly lower daily average temperature measured inside the mesocosms. There was no consistent difference in temperature between mesocosms with different lid types. Water content did not differ between mesocosms with open and closed lids, or between samples with and without mesocosms at the end of the experiment (Supplementary information, Table S2).

## Discussion

Regulatory policies aim to minimise the footprint of anthropogenic activities on ecosystems. However, the effects of such activities, e.g., insecticide application, on soil biodiversity and community composition, have not yet been incorporated thoroughly in regulatory decision-making. This can largely be attributed to the complex interaction and high uncertainty in the effects at the field community level (Bach et al., 2020). With the current two-tier risk assessment of pesticides in soil, a field test must be performed if the standard toxicity tests with earthworms, predatory mites and springtails do not provide sufficient evidence for pesticide safety in soil (EFSA Panel on Plant Protection Products and their Residues (EFSA PPR 2017)). However, an intermediate tier using field mesocosms such as described here, could be used to obtain additional information about toxicity at the community level with lower cost and labour.

Using mesocosms with intact soil cores in the field allowed us to assess the effects of imidacloprid on the abundance of soil arthropod fauna in their natural habitat, as well as their potential migration. Our experimental setup is a robust method for assessing the effects of pesticide exposure on natural soil fauna abundance under field-realistic conditions, as the mesocosm enclosure of the soil core does not change the natural environment beyond the effects of the pesticide being assessed. Temperature and moisture influence the toxicity of imidacloprid in the springtail *Folsomia candida* (Bandeira et al., 2020; Braúlio Hennig et al., 2020). Although the soil moisture content did not differ between undisturbed soil and soil in mesocosms, the temperature outside our mesocosms was ~1°C higher during the day and lower during the night inside mesocosms compared to outside.,. However, this difference is most likely not large enough to influence the abundance of soil fauna.

The higher imidacloprid concentrations reduced the abundance of springtails substantially, whereas mite abundance remained unaffected. This is consistent with other studies indicating that predatory and oribatid mites tolerate exposure to neonicotinoids better than springtails (Cheng et al., 2021; de Lima e Silva et al., 2017). The log-linear models explained a lot of the variance in the data for the springtails but not for the mites. In line with this, statistical power was much lower for the models fitted to mites. This supports our findings that imidacloprid had very little or no effect on mites.

Imidacloprid is a highly water-soluble compound that easily diffuses through the ventral tube of the springtails, making soil pore water the major exposure route for these organisms (Ogungbemi and van Gestel, 2018). Pore water is unlikely to diffuse into the mites due to the absence of a ventral tube and the presence of a resistant cuticle. Therefore, the main exposure route is most likely through their food (Natal-da-Luz et al., 2019), which would imply that a longer experimental time would be necessary for the added imidacloprid to be taken up and redistributed before an effect could be measured.

Although declines in abundance were evident in both surface- and soil-dwelling springtails, a concentration-dependent decline was evident only in surface-dwelling springtails. Notably, the mesocosms contained many more surface-living springtail species than soil-living ones, implying that the high variation in the samples becomes more influential for the soil-dwelling springtails. Heterogeneity in soil conditions across both horizontal and vertical scales is typically encountered under field conditions. The surface-living springtails are likely to have encountered higher concentrations than the soil-living springtails because imidacloprid was applied to the surface. Chemical analysis of the soil layers in a similar mesocosm study at the same field site confirms this assumption, but also suggests that under the influence of rainfall a considerable amount of imidacloprid added to the surface will distribute to deeper layers in the soil column over time (Teksum, 2021). The latter would also be expected considering the high water solubility and associated high mobility of imidacloprid in soil.

The LC50 values for surface-dwelling springtails were comparable to or somewhat lower than those reported for the soil-dwelling model springtail *Folsomia candida* commonly used in standard OECD and ISO springtail tests for soil quality (de Lima e Silva et al. 2017, 2020, 2021). However, springtail species more often found in natural habitats showed higher tolerance to neonicotinoid insecticides than *F. candida* (de Lima e Silva et al., 2021). From the present study, differences between surface- and soil-dwelling springtail species seen already after a short exposure time suggest that there are some differences between the responses of species and/or functional groups. Underlying mechanisms for these differences would be important to identify for improving our understanding of long-term exposure effects. So far, we have an indication that surface dwelling springtail species are more sensitive than soil living species at high exposure concentrations. A longer-lasting study may clarify the differences in responses of these two groups of springtails.

Allowing or limiting migration using open or closed lids, respectively, did not modulate the abundance of springtails or mites, suggesting a low effect of animal movements in and out of the mesocosms. However, some interesting tendencies were found. For instance, the effect sizes suggested that although not statistically significant, all taxa except for the saprotrophic mites tended to be more abundant in the imidacloprid-exposed mesocosms with open lids (Table 1). A plausible explanation for this tendency may be a replacement of dead mesofauna by immigration from the surrounding field. As imidacloprid acts as an acetylcholine agonist, it is likely that migration may be affected through paralysis or avoidance behaviour, although this was not investigated in the present study (Alves et al., 2013; Bernardino et al., 2021; Ge et al., 2018; Larink and Sommer, 2002; Zaller et al., 2016). The importance of dispersal and migration may vary between species, habitat and litter type, and season (Janion-Scheepers et al., 2016; Widenfalk et al., 2018). Thus, a longer-lasting experiment starting early in the growing season when imidacloprid is usually applied, including the natural variation in climate and phenology, is likely to provide more information about how animal vagility may affect responses to imidacloprid. Many responses linked to life history traits take a much longer time than the duration of the present study for a detailed assessment, and sensitivity may differ across developmental stages (Baines et al., 2017; Guimaraes et al., 2019; Tran et al., 2020). However, a small but significantly lower number of predatory mites was found in the mesocosm samples compared to the undisturbed samples (Table 2). This may suggest that also the lids with a large mesh size may have influenced the movement of predatory mites. Other mechanisms determining the effects of migration and competition cannot be ruled out based on the present study.

In conclusion, using mesocosms with intact soil cores in the field is a feasible way to investigate soil community responses to a chemical stressor. Variation in responses among the different functional groups and taxa with different habitat preferences is expected and should be accounted for during risk assessments, for instance when evaluating different pesticide application practices.

## Supplementary information

Supplementary Information

## Data Availability

The datasets generated during the current study are openly available in the DataverseNO repository, 10.18710/QWIDIT.
