# Supplementary material for: Pesticide effects on the abundance of springtails and mites in field mesocosms at an agricultural site
Source: Ecotoxicology. 2022 Nov 1;31(9):1450–61. doi: 10.1007/s10646-022-02599-3 (PMC9652236; doi:10.1007/s10646-022-02599-3)
Supplement: Supplementary file 1 — Supplementary Information [file 10646_2022_2599_MOESM1_ESM.pdf]

Pesticide effects on the abundance of springtails and mites in field mesocosms at an agricultural site

Heidi Sjursen Konestabo[1,2], Tone Birkemoe[3], Hans Petter Leinaas[1], Cornelis A.M. van Gestel[4], Sagnik Sengupta[1], Katrine Borgå[1]

[1]Department of Biosciences, University of Oslo, Norway;

[2]The Science Library, University of Oslo, Norway;

[3]Faculty of Environmental Sciences and Natural Resource Management, Norwegian University of Life Sciences, Norway;

[4]Amsterdam Institute for Life and Environment (A-LIFE), Faculty of Science, Vrije Universiteit, Amsterdam, The Netherlands

Supplementary information

Data availability statement: The datasets generated during the current study are openly available in the DataverseNO repository, https://doi.org/10.18710/QWIDIT

Table S1: Total number of springtails and mites in soil samples from field mesocosms treated with imidacloprid, and untreated samples at the start and end of the experiment (n=60). Functional groups are based on microhabitat (springtails) or trophic level (mites). At the field site surface-living (epedaphic + hemiedaphic) springtails were dominated by Hypogastruridae and Isotomidae, and a few Symphypleona and Entomobryidae were found. Soil-living springtails (euedaphic) were dominated by Onychiuridae, mostly from the genus *Mesaphorura*. Most mites belonged to the saprotrophic Oribatida. Free-living Prostigmata, also assumed to be saprotrophic, were present in high numbers in some samples. The predatory Mesostigmata were mostly large surface-living Gamasina.

Taxa	Total number of individuals	Estimated number of individuals /m^2	Functional group
All springtails	7390	62730	
Hypogastruridae	1587	13471	Epedaphic / hemi-edaphic
Isotomidae	5309	45066	Epedaphic / hemi-edaphic
Entomobryidae	75	637	Epedaphic / hemi-edaphic
Onychiuridae	388	3294	Euedaphic
Symphypleona	31	263	Epedaphic / hemi-edaphic
All mites	5885	49956	
Oribatida	3539	30041	Saprotrophic
Mesostigmata	650	5518	Predatory
Prostigmata	1696	14397	Saprotrophic

Table S2: Average soil moisture content of mesocosms and undisturbed soil samples taken from a mesocosm study in an agricultural field to assess the effects of imidacloprid on soil micro-arthropod communities. Soil moisture content is given as percentage of soil fresh weight in the different samples.

**Sample type**	**Average soil moisture content (%)**	**±SE**
Mesocosms with open lids	37.5	2.1
Mesocosms with closed lids	37.4	3.8
Undisturbed samples	36.5	1.4

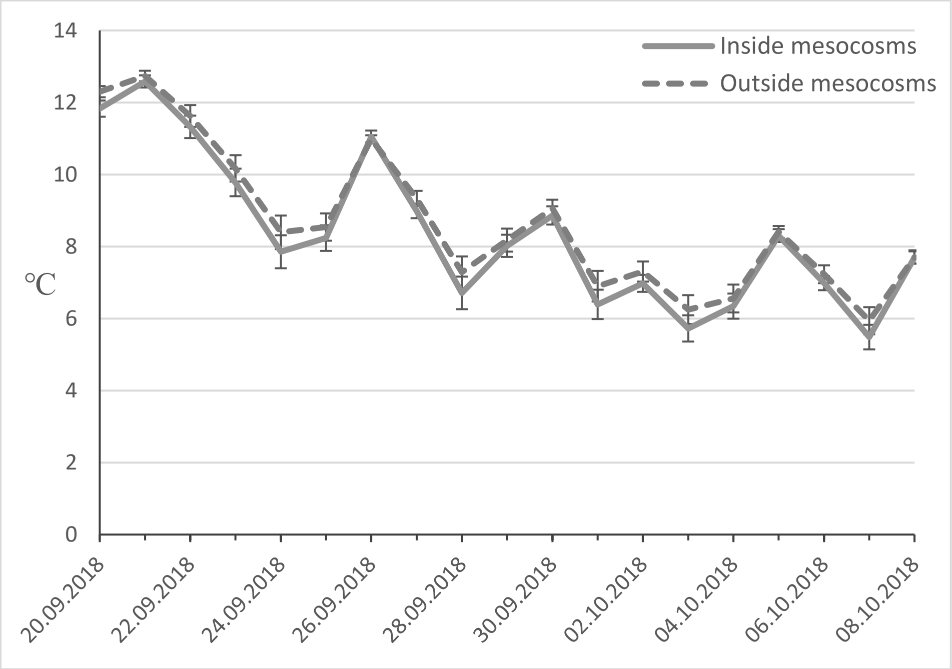

Fig. S1. Daily average (±SD) temperatures measured inside (solid line) and outside field-incubated mesocosms (dashed line), at a depth of 0.5-1 cm into an agricultural soil. Mesocosms with different lid types (open or closed lids) are pooled (n=6).
